# Water Oxidation with Cobalt‐Loaded Linear Conjugated Polymer Photocatalysts

**DOI:** 10.1002/anie.202008000

**Published:** 2020-08-19

**Authors:** Reiner Sebastian Sprick, Zheng Chen, Alexander J. Cowan, Yang Bai, Catherine M. Aitchison, Yuanxing Fang, Martijn A. Zwijnenburg, Andrew I. Cooper, Xinchen Wang

**Affiliations:** ^1^ Department of Chemistry and Materials Innovation Factory University of Liverpool Liverpool L7 3NY UK; ^2^ Department of Pure and Applied Chemistry University of Strathclyde Thomas Graham Building 295 Cathedral Street Glasgow G1 1XL UK; ^3^ State Key Laboratory of Photocatalysis on Energy and Environment College of Chemistry Fuzhou University Fuzhou 350116 P. R. China; ^4^ Stephenson Institute for Renewable Energy University of Liverpool Chadwick Building Peach Street Liverpool L69 7ZF UK; ^5^ Department of Chemistry University College London 20 Gordon Street London WC1H 0AJ UK

**Keywords:** oxygen production, polymer photocatalysts, soft photocatalysts, solar fuel, water oxidation

## Abstract

The first examples of linear conjugated organic polymer photocatalysts that produce oxygen from water after loading with cobalt and in the presence of an electron scavenger are reported. The oxygen evolution rates, which are higher than for related organic materials, can be rationalized by a combination of the thermodynamic driving force for water oxidation, the light absorption of the polymer, and the aqueous dispersibility of the relatively hydrophilic polymer particles. We also used transient absorption spectroscopy to study the best performing system and we found that fast oxidative quenching of the exciton occurs (picoseconds) in the presence of an electron scavenger, minimizing recombination.

## Graphical abstract

Ten linear conjugated organic polymer photocatalysts are presented for water oxidation in the presence of an electron scavenger. The results can be explained by a combination of factors, such as thermodynamic driving force, light absorption, and dispersibility of the relatively hydrophilic polymer particles. Transient absorption spectroscopy was used to study the charge‐carrier dynamics.

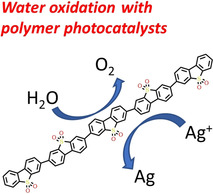

## Introduction

Photocatalytic water splitting has the potential to generate storable fuel from a renewable resource without side‐products that contribute to climate change.^1^, ^2^ A large number of inorganic semiconductors has been studied as photocatalysts for sacrificial half reactions that produce either hydrogen or oxygen in the presence of hole or electron scavengers.^1^, ^3^ This has resulted in systems that perform overall water splitting with promising solar‐to‐hydrogen efficiencies.^4^, ^5^, ^6^, ^7^

Organic photocatalysts, while topical, are much less widely explored; carbon nitride is by far the best studied system since the first report as a photocatalyst in 2009.^8^ There has been growing interest recently in other conjugated organic materials that can be synthesized using cross coupling or condensation reactions,^2^ such as conjugated microporous polymers (CMPs),^9^, ^10^, ^11^, ^12^, ^13^ linear conjugated polymers,^14^, ^15^, ^16^, ^17^, ^18^, ^19^, ^20^, ^21^, ^22^ covalent organic frameworks,^23^, ^24^, ^25^, ^26^ and triazine‐based frameworks.^27^, ^28^, ^29^, ^30^ Many of these systems have shown good photocatalytic performance for hydrogen production from water in the presence of a sacrificial hole scavenger.^2^ Ultimately, however, we need to develop systems that do not rely on sacrificial scavengers. To achieve this, a wider range of materials that drive water oxidation is required. Besides carbon nitride, a small number of covalent triazine‐based frameworks,^27^, ^28^, ^29^, ^30^ covalent organic frameworks,^31^ and CMPs^32^, ^33^ have been reported to facilitate water oxidation after loading with metal co‐catalysts, while poly(benzimidazobenzophenanthroline) is a rare example of a photoanode for water oxidation.^34^ There are strong drivers to diversify this small range of organic photocatalysts for water oxidation and, particularly, to develop materials that function under sunlight; that is, materials that absorb visible rather than only UV light. For example, this could allow us to construct all‐organic Z‐schemes that comprise an organic proton reduction catalyst coupled with an organic water oxidation catalyst.

## Results and Discussion

**Figure 1:**
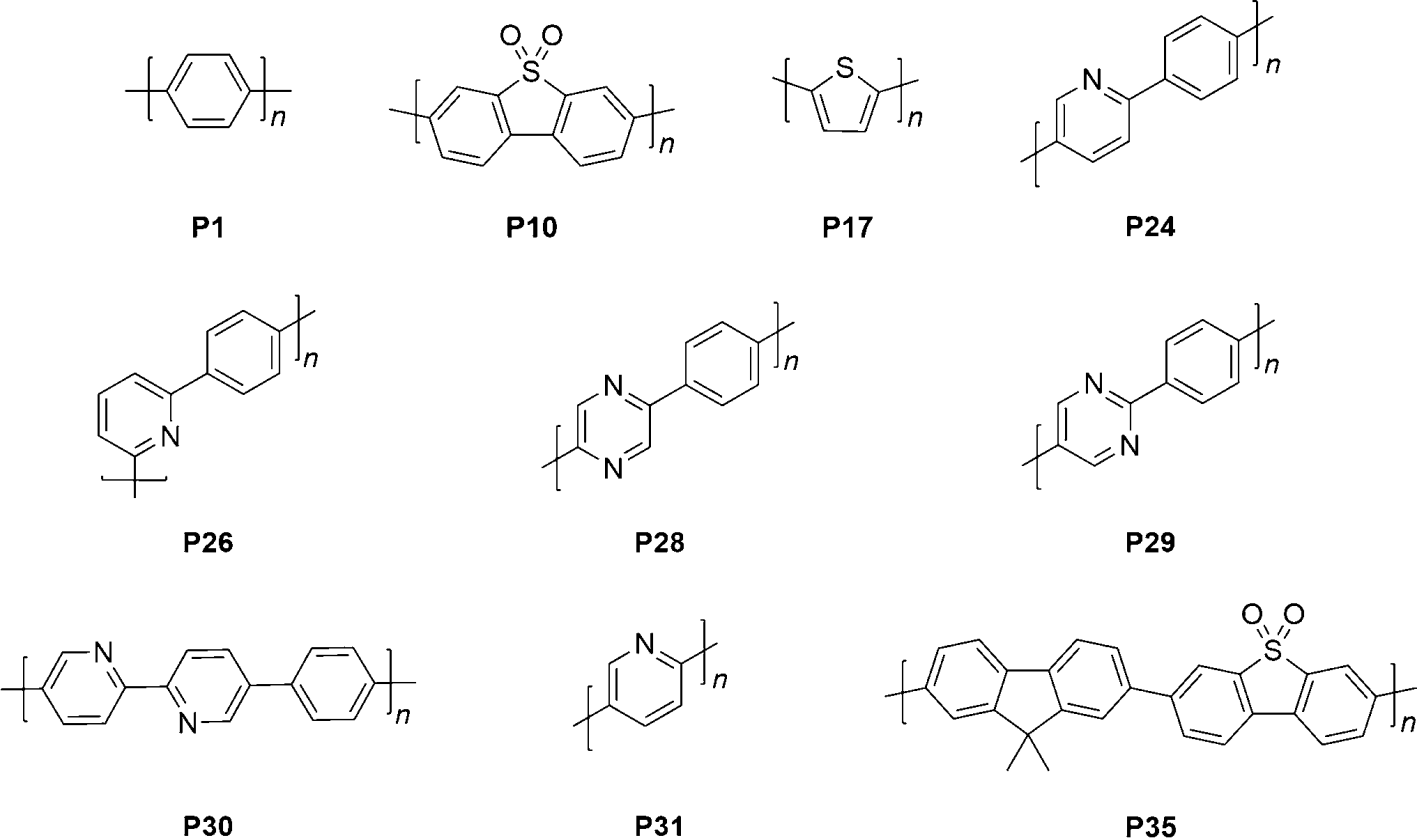
Structures of the 10 linear polymer photocatalysts investigated in this study for water oxidation.

**Figure 2:**
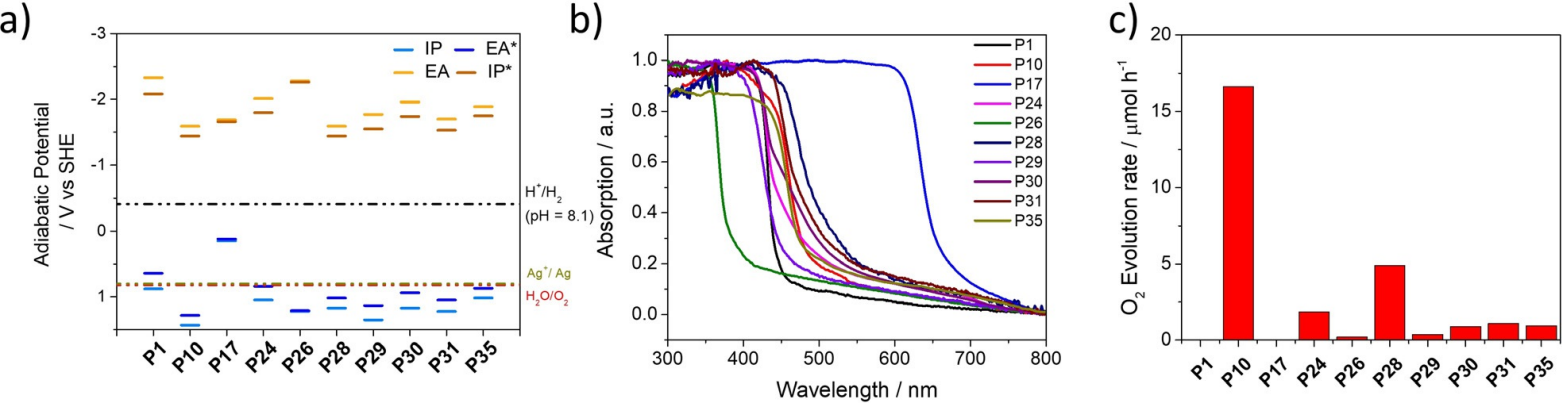
a) TD‐B3LYP predicted potentials of the charge carriers (IP, EA) and excitons (IP*, EA*) in the polymer photocatalysts (values taken from refs.^16^, ^20^, ^22^). b) UV/Vis spectra of all polymer photocatalysts measured in the solid‐state. c) Photocatalytic oxygen evolution of all polymer photocatalysts under broadband illumination (full arc, 300 W Xe light source). Conditions: Polymers (50 mg) loaded with 1 wt. % cobalt, water (100 mL), AgNO_3_ (0.01 m), La_2_O_3_ (200 mg).

Here, we study a range of cobalt‐loaded linear conjugated polymer photocatalysts for oxygen evolution from water (Figure 1). This is the first time that linear conjugated polymers have been reported to photocatalyze this challenging reaction. We studied ten polymers, all of which, with the exception of P1 and P17 (Figure 2 a), were predicted by previous DFT calculations to have the necessary driving force for water oxidation.^16^, ^20^, ^22^, ^35^ All polymers were made using Pd^0^ catalyzed Suzuki–Miyaura polycondensation reaction of dibromo arenes with diboronic acids/ acid ester arenes except for P17, which was made using Stille coupling of distannyl and dibromo thiophene (see ESI for experimental details). These polymers were loaded with a co‐catalyst via photo deposition of a cobalt species prior to the catalysis experiments.

The activity of the cobalt‐loaded polymer photocatalysts (50 mg in 100 mL water)for water oxidation was measured under broadband (full arc, 300 W Xe light source) and visible (*λ*>420 nm, 300 W Xe light source) irradiation in the presence of AgNO_3_, which acts as the electron scavenger, and La_2_O_3_ as a pH buffer. The photophysics of the best‐performing photocatalyst, P10, were also studied by time‐correlated single photon counting and transient absorption spectroscopy, both before and after the addition of the cobalt co‐catalyst and the AgNO_3_ solution. Apart from poly(*p*‐phenylene) (P1) and poly(thiophene) (P17), the two materials predicted to lack a thermodynamic driving force (Figure 2 a), all polymers acted as photocatalysts for water oxidation under broadband irradiation to some extent. The oxygen evolution rates (OERs) for the 8 photoactive polymers spanned a significant range (Figure 2 c). A maximum OER of 16.6 μmol h^−1^ was observed for poly(dibenzo[*b*,*d*]thiophene sulfone) (P10), while the OER for the *meta*‐linked co‐polymer of pyridine‐phenylene, P26, was just 0.2 μmol h^−1^.

The variation in the water oxidation performance can be rationalized, as previously for sacrificial hydrogen evolution rates,^21^, ^36^ by differences in the potentials of the charge carriers, the optical gap of the polymers, and the dispersibility of the materials in water. P1 and P17 were predicted to have the shallowest, least positive ionization potentials relative of the polymers studied here. Hence, these materials lack the thermodynamic driving‐force for water oxidation at pH 8.1, which was the experimental pH of the AgNO_3_–La_2_O_3_ solution. Neither material produces any oxygen under these experimental conditions. By contrast, P10 has the deepest, most positive, ionization potential (Figure 2 a) and it is also the best‐performing photocatalyst, evolving 16.6 μmol h^−1^ under broadband irradiation (full arc, 300 W Xe light source). Photocatalyst P10 performs significantly better than its fluorene co‐polymer analog P35^13^ (OER=1.0 μmol h^−1^), which can be explained by a loss in driving‐force combined with the much poorer aqueous dispersibility of P35 compared to P10. This was quantified by light obscuration measurements of the photocatalyst particles dispersed in water/AgNO_3_ whereby a low transmission value (*T*; see Table 1) corresponds to an opaque suspension where the particles are well dispersed.^21^ For P10, the transmission was determined to be very low, with a value of 0.4 %, because it is the most dispersible polymer in the study; this can be compared with a transmission value of 56.3 % for P35, which is one of the least dispersible polymers considered.

**Table 1:** Optical gap, band positions, optical transmissions, and oxygen evolution rates (OERs) for the 10 polymer photocatalysts.

Photocatalyst	Optical gap^[a]^ [eV]	IP vs. SHE^[b]^ [V]	EA vs. SHE^[b]^ [V]	*T* ^[c]^ [%]	OER^[d]^ [μmol h^−1^]
P1	2.78	0.88	−2.33	59.1	0
P10	2.62	1.43	−1.59	0.4	16.6
P17	1.89	0.15	−1.69	73.7	0
P24	2.76	1.05	−2.01	4.5	1.9
P26	3.22	1.22	−2.28	1.3	0.2
P28	2.45	1.17	−1.59	11.3	4.9
P29	2.73	1.35	−1.77	37.8	0.4
P30	2.72	1.17	−1.96	55.2	0.9
P31	2.51	1.22	−1.70	45.8	1.1
P35	2.59	1.02	−1.89	56.3	1.0

[a] Calculated from the on‐set of the absorption spectrum; see the discussion in the Supporting Information. [b] Predicted using (TD‐)DFT (values taken from refs^16^, ^20^, ^22^). [c] Average optical transmission of the polymer dispersed in water/AgNO_3_. [d] Reaction conditions: 50 mg polymer photocatalysts loaded with cobalt was suspended in water/AgNO_3_/La_2_O_3_, 300 W Xe light source full arc irradiation.

The nitrogen‐containing polymers, P24, P25, and P28–P31, all acted as photocatalysts under broadband illumination, albeit with much lower activities than for P10. As for observations that we made for hydrogen production,^20^ we found the highest rate in among the nitrogen‐containing polymers for the pyrazine‐*co*‐phenylene polymer (P28), with an OER of 4.9 μmol h^−1^ under broadband irradiation (full arc, 300 W Xe light source). Photocatalyst P26 has an ionization potential that is similar to P28 and is even more dispersible in water, with a transmission value of 1.2 % compared to 11.3 % for P28 (see Table 1). However, the *meta*‐linkage results in a blue‐shifted absorption on‐set, thus limiting the performance to 0.2 μmol h^−1^ because it absorbs less light. The same detrimental effect of the introduction of 1,3‐linkages in polymers on their performance as photocatalysts was previously observed by us for hydrogen evolution.^13^, ^20^ Photocatalyst P24, which contains pyridine, has a OER of 1.9 μmol h^−1^; it is slightly more dispersible in water than P28 with a transmission value of 4.5 %, but it also has a slightly lower driving force for water oxidation and a considerably larger optical gap again limiting the amount of light that is absorbed and thus the amount of holes available for OER. Finally, P29–P31 are significantly less dispersible in water than P10, P24, P26 and P28, with transmission values ranging from 37.8 % to 56.3 %, which most likely explains their lower OERs (0.4–1.1 μmol h^−1^).

The dispersibility of the different polymers depends both on their wettability (Supporting Information, Figures S5, S6, Table S1) and, to some extent, the particle‐size distribution (Supporting Information, Figures S10, S12, Table S2). This is the reason that polymers such as P10 and P24 containing hydrogen bond acceptors, such as sulfone groups and pyridinic nitrogen, are on average more dispersible in water than polymers that lack these groups, such as P1 and P17. We note that water oxidation using a water‐soluble inorganic electron scavenger, AgNO_3_, is more challenging in terms of polymer dispersibility than for hydrogen evolution because there is no organic component, such as triethylamine or triethanolamine, which can help to disperse these conjugated polymers in water. This is a particular issue with hydrophobic polymers. By extension, this will be an important consideration for overall water splitting, where no sacrificial agents are present.

The excited state lifetime of the polymers, as studied by time correlated single photon counting in the solid‐state (Supporting Information, Table S3), showed no clear correlation with the observed oxygen evolution rates (Supporting Information, Figure S18), but the reduction in lifetime was largest for P10 when comparing materials before and after cobalt loading (2.78 vs. 1.41 ns). As expected, we observed that the addition of AgNO_3_ results in a reduction in the exciton lifetime for cobalt‐loaded P10 when measured in suspension (Supporting Information, Figure S16).

**Figure 3:**
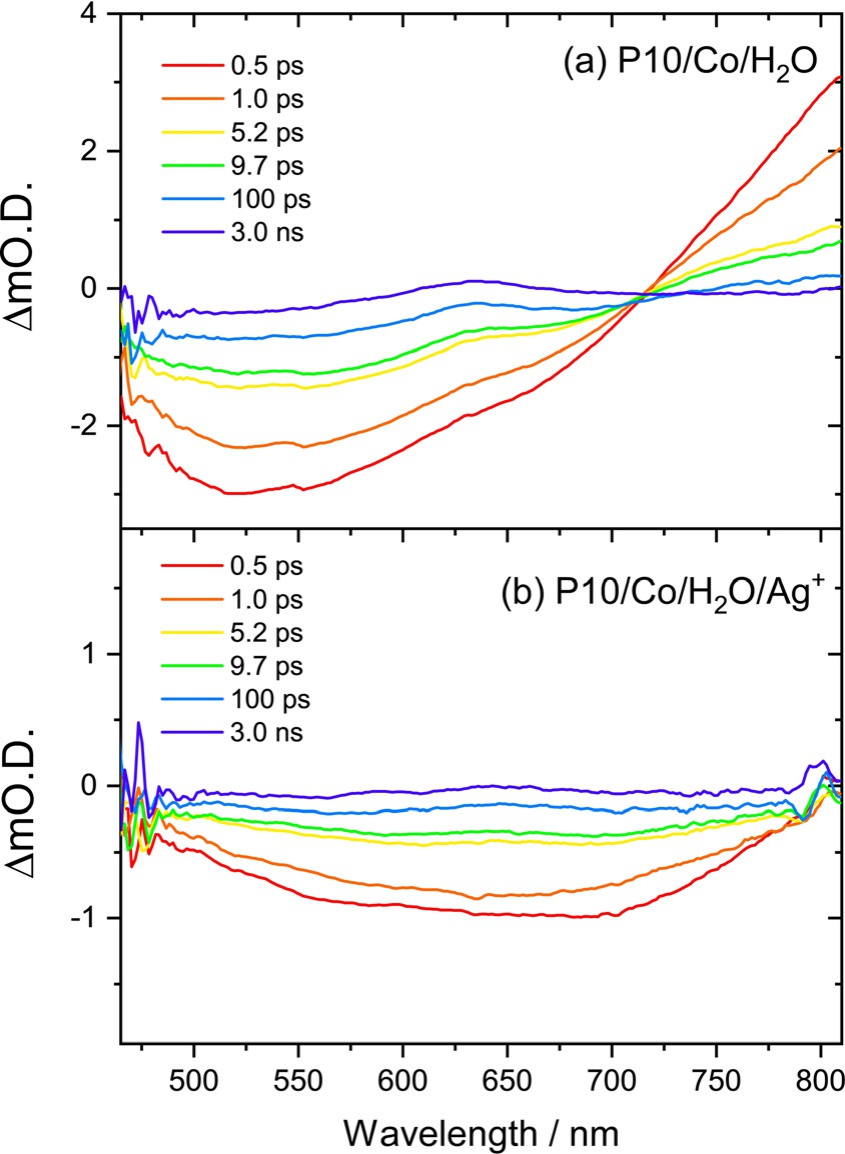
a) Transient absorption spectra of P10 loaded with 1 wt. % cobalt in water and b) AgNO_3_ (0.01 m) following 400 nm (150 nJ pulse, 5 kHz) excitation.

Transient absorption (TA) spectroscopy was used to study the effect of the cobalt co‐catalyst and Ag^+^ scavenger on the dynamics of the photogenerated charge carriers of P10. The TA spectra of P10 with cobalt present in pure water following 400 nm excitation (Figure 3 a) showed the same behavior as observed previously for P10 in the absence of cobalt^16^, ^37^ (Supporting Information, Figure S19). The broad negative signal from <465 to 705 nm was previously assigned to stimulated emission with the excited state absorption from 705 to >810 nm due to singlet exciton formation. We propose the same assignment for P10/Co (Figure 3 a), demonstrating that exciton quenching by charge transfer to the cobalt co‐catalyst when in an aqueous suspension is not a significant pathway. This conclusion is supported by the minimal change in steady‐state PL of P10 samples with and without cobalt in water (Supporting Information, Figure S20) and the only slight change in lifetime measured by time correlated single photon counting (Supporting Information, Table S3).

For both P10 (Supporting Information, Figure S19) and P10/Co (Figure 3 a) at longer time scales (>1 ns), the stimulated emission and exciton photoinduced absorption bands decay and a weak, positive band at 637 nm remains. Studies on the microsecond to seconds timescale with P10 in the presence of a sacrificial hole scavenger assign this band to an electron polaron. For P10 in water alone on the ultrafast timescale, the 637 nm band was also observed to grow in within 1–2 ps, leading to an assignment of polaron pair that has spectral characteristics that are similar to the fully separated electron.^16^

Addition of Ag^+^ to the P10/Co sample caused rapid quenching of the exciton, as demonstrated by the complete lack of stimulated emission and the loss of the excited state absorption band at >705 nm (Figure 3 b). Instead, a broad bleach between 460 to 800 nm was present, which recovered by 3 ns.

The broad bleach is due to the ground‐state of P10, which in the presence of Ag^+^ shows a shift in UV/Vis maxima and the formation of a broad shoulder across the visible region in the UV/vis absorption spectrum, centered around 500 nm (Supporting Information, Figure S22). In the presence of Ag^+^, the long‐lived 637 nm band, assignable either to a partially separated state or an electron polaron, is also absent (Supporting Information, Figures S23, S24). It is clear that Ag^+^, a commonly used electron scavenger, is preventing the formation of long‐lived photogenerated electrons, most likely through oxidative quenching of the exciton. It is striking that we do not observe spectral features owing to exciton formation even at the very early (0.5 ps) timescales studied and a degree of pre‐association between P10/Co and Ag^+^, indicated by the change in ground state UV/vis absorption spectrum, may be a crucial factor in enabling efficient electron scavenging. We cannot detect spectral features assignable to holes on the P10 or P10/Co samples by TA spectroscopy in the visible region. Previous spectroelectrochemical studies of cobalt‐based water oxidation catalysts reported that the Co^III/IV^ species formed during water oxidation have featureless UV/Vis absorption spectra in the region of study here, thus making it difficult to address the timescale of water oxidation and hole transfer to the co‐catalyst.^38^

The rate of 16.6 μmol h^−1^ under broadband irradiation for P10 loaded with 1 wt % cobalt via photodeposition, the best‐performing photocatalyst in this study, was reduced to 5.2 μmol h^−1^ under visible light irradiation, which is still an appreciable rate. Indeed, these rates are higher than for a previously reported cobalt‐loaded biphenyl‐linked triazine‐based framework, CTP‐2, with rates of approximately 1.5 μmol h^−1^ under visible light and 3 μmol h^−1^ under broadband illumination when measured on exactly the same set‐up that we used here.^29^ This like‐with‐like comparison is important because the rates for these photocatalytic reactions depend strongly on the precise experimental set‐up and the light source used.^39^ In this context, it is important to note that the absolute rate of CTP‐2 is not the highest reported to date,^30^, ^32^ but this material can be made in a simple one‐step reaction, which makes it a useful benchmark for comparison across different experimental photolysis set‐ups. The rate under visible light is somewhat lower than well‐studied inorganic photocatalysts BiVO_4_ and WO_3_ under visible light irradiation with approximate rates of 13 and 10 μmol h^−1^, respectively.^40^

We went on to test P10 loaded with different amounts of cobalt but found no improvement: the initial loading of 1 wt % Co^2+^ resulted in the highest photocatalytic activity (Supporting Information, Figure S25). As‐prepared P10, which contains residual palladium (0.33 wt %) originating from the polymer synthesis, gave a lower but measurable oxygen evolution rate of 1.20 μmol h^−1^ under broadband irradiation (full arc, 300 W Xe light source) and 0.95 μmol h^−1^ under visible light irradiation (*λ*>420 nm, 300 W Xe light source). Sulfones^41^ as well as pyridines^42^ can act as ligands for cobalt, which might also impact the photocatalytic performance of the materials in this study. X‐Ray absorption spectroscopy of P10/Co (Supporting Information, Figure S27) indicates that cobalt is, similar to other reports,^43^, ^44^ present as CoO_*x*_, which is believed to be the active species for water oxidation.

Cobalt‐loaded P10 was also found to be active with sodium persulfate and FeCl_3_ electron scavengers under broadband illumination, giving rates of 3.8 μmol h^−1^ and 11.1 μmol h^−1^, respectively. FeCl_3_ is of particular interest because it possible to reoxidize the product of the reaction, Fe^2+^, with a hydrogen evolution catalyst that again produces Fe^3+^, thus potentially acting as a mediator in a Z‐Scheme to facilitate overall water‐splitting.^45^ This is not possible for metallic silver and is the product of the AgNO_3_ scavenger.

**Figure 4:**
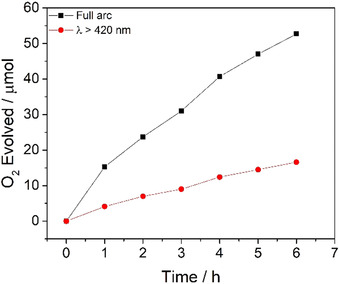
Photocatalytic oxygen evolution of photocatalyst P10 under broadband (full arc, 300 W Xe light source) and visible‐light illumination (*λ*>420 nm, 300 W Xe light source). Conditions: Photocatalyst P10 (50 mg) loaded with 1 wt % cobalt, water (100 mL), AgNO_3_ (0.01 m), La_2_O_3_ (200 mg).

Experiments in the absence of photocatalyst showed that no oxygen production occurred (Supporting Information, Figure S1) and the photocatalytic stability of P10 was evaluated using both broadband (full arc, 300 W Xe light source) and visible light irradiation (*λ*>420 nm, 300 W Xe light source; Figure 4). In both cases, the OER decreases over time because the material is increasingly covered with metallic silver, which is the side product of the water oxidation. This results in shadowing of the sample, as evident in bright field and high‐angle annular dark field STEM imaging (Supporting Information, Figure S28).^29^ Likewise, we observe that the material is no longer fluorescent (Supporting Information, Figure S29) and the UV/Vis spectrum (Supporting Information, Figure S30) also indicates deposition of silver on the material. Further characterization via FTIR (Supporting Information, Figure S31) shows no changes that can be related to decomposition of the photocatalyst, although it is possible that the silver coating also protects the polymer from the light. Nonetheless, these data suggest that silver deposition, rather than auto‐oxidation of the polymer as a result of the build‐up of holes, is responsible for the loss of OER activity, at least on the timescales of these experiments.

## Conclusion

We have demonstrated the first use of cobalt‐loaded conjugated linear organic polymers as photocatalysts for water oxidation. Among ten systems studied, P10, a dibenzo[*b*,*d*]thiophene sulfone homopolymer, was the best‐performing material after photo‐deposition of a cobalt co‐catalyst, giving oxygen evolution rates that significantly exceed those observed for related triazine‐based frameworks under identical experimental conditions. The relative oxygen evolution activity of the polymers can be understood in terms of their predicted ionization potentials, which control the driving force for water oxidation, along with the optical gap and the aqueous dispersibility of the polymers. The latter is particularly important for water oxidation since unlike for sacrificial hydrogen production, there are no organic scavengers, such as aliphatic amines, to help to disperse the polymers in water.

P10 has the largest driving force for water oxidation amongst the materials tested; it is also the most dispersible in water and has a relatively low optical gap. Materials that were predicted to lack the required driving force for water oxidation did not oxidize water, suggesting a degree of a priori designability for these materials. Transient absorption spectroscopy was used to study the charge‐carrier dynamics of P10 to understand the underlying kinetic processes. This study lays the groundwork for overall water splitting in an all‐organic catalyst system: for example, by combining two polymer photocatalysts, one for proton reduction and one for water oxidation.

## Conflict of interest

The authors declare no conflict of interest.

## Supporting information

As a service to our authors and readers, this journal provides supporting information supplied by the authors. Such materials are peer reviewed and may be re‐organized for online delivery, but are not copy‐edited or typeset. Technical support issues arising from supporting information (other than missing files) should be addressed to the authors.

Supplementary
