# Supplementary material for: Water Oxidation with Cobalt‐Loaded Linear Conjugated Polymer Photocatalysts
Source: Angew Chem Int Ed Engl. 2020 Aug 19;59(42):18695–700. doi: 10.1002/anie.202008000 (PMC7589379; doi:10.1002/anie.202008000)
Supplement: Supplementary file 1 — Supplementary [file ANIE-59-18695-s001.pdf]

Angewandte Chemie
Eine Zeitschrift der Gesellschaft Deutscher Chemiker

Supporting Information

Water Oxidation with Cobalt-Loaded Linear Conjugated Polymer Photocatalysts

Reiner Sebastian Sprick, Zheng Chen, Alexander J. Cowan,* Yang Bai, Catherine M. Aitchison, Yuanxing Fang, Martijn A. Zwijnenburg,* Andrew I. Cooper,* and Xinchen Wang**

anie_202008000_sm_miscellaneous_information.pdf

Experimental Procedures

General methods. Photocatalysts P1,[1] P10,[2] P17,[3] P24, P26, P28, P29, P30, P31,[4] P35[5] were synthesized using previously reported procedures. All other reagents were obtained from Sigma-Aldrich, or Fluorochem and used as received. ICP-MS analysis was performed on a Perkin Elmer ICP MS NexION 2000 with equipped with a collision/reaction cell after a microwave digest of the materials in nitric acid (67-69%, trace metal analysis grade) in a microwave. The solutions were diluted with water before the measurement and the instrument was calibrated with Co standards in aqueous solution and Y-89 as the internal standard. Transmission FT-IR spectra were recorded on a Bruker Tensor 27 at room temperature; samples were prepared as pressed KBr pelletsThe UV-visible absorption spectra of the polymer networks were recorded on a Shimadzu UV-2550 UV-vis spectrometer as powders in the solid state. The fluorescence spectra of the polymer powders were measured with a Shimadzu RF-5301PC fluorescence spectrometer at room temperature in the solid state. TCSPC experiments were performed on an Edinburgh Instruments LS980-D2S2-STM spectrometer equipped with picosecond pulsed LED excitation sources and a R928 detector, with a stop count rate below 3%. An EPL-375 diode (λ = 370.5 nm, instrument response 100 ps, fwhm) was used as the light source. Suspensions were prepared by ultrasonicating the polymer in water. The instrument response was measured with colloidal silica (LUDOX HS-40, Sigma-Aldrich) at the excitation wavelength. Decay times were fitted in the FAST software using suggested lifetime estimates. Imaging of the polymer morphology was achieved on a Hitachi S4800 Cold Field Emission SEM, with secondary electron, backscatter and transmission detectors. Water contact angles were measured on a drop-shape analysis apparatus (Krüss DSA100) dosing 5 µL drops of water onto the surface of pressed pallets of the photocatalysts. Contact angles were fitted using the Young–Laplace equation. X-Ray photoelectron spectroscopy was measured on a Thermo Fisher ESCALAB 250 XPS spectrometer with a monochromatized Al Kα line source (200 W) using a sample of P10 after photo-deposition of Co from $Co(NO_3)_2$. Zeta-potential were measured by Malvern Zeta Sizer Nano-ZS90. The samples were prepared by suspending 10 mg of the samples in 10 mL deionized water. Static light scattering measurements were performed on a Malvern Mastersizer 3000 Particle Sizer, polymers were dispersed in water by ultrasonication for 10 minutes and the resultant suspensions were injected into a stirred Hydro SV quartz cell, containing water, to give a laser obscuration of 2-8%. Particle sizes were fitted according to Mie theory, using the Malvern 'General Purpose' analysis model, for non-spherical particles with fine powder mode turned on. A polymer refractive index of 1.59, polymer absorbance of 0.1 and solvent refractive index of 1.330 were used for fitting. Sauter mean diameter *D*[3,2] gives the diameter of a sphere that has the same volume-to-surface-area ratio as the entire distribution calculated using Equation 1.[6,7]

$$\boldsymbol{D[3,2]} = \frac{\sum_1^n D_{iv_i}^3}{\sum_1^n D_{iv_i}^2} \qquad \textbf{Equation 1}$$

Transmittance of the polymer photocatalysts in water dispersion was measured on a Formulaction S.A.S. Turbiscan AGS system with an 880 nm NIR diode and a detector at 180° (relative to the light source) in a cylindrical glass cell. Samples were prepared by dispersing the photocatalysts in 5 mL water and then diluting with water up to 30 mL total volume. All samples were sonicated before each measurement.

Co-catalyst loading. Each sample of photocatalyst (100 mg) was dispersed in a mixture of water (100 mL) and methanol (10 mL) by ultrasonication for 10 minutes. $Co(NO_3)_2$ was added and the mixture was then illuminated with a 300 W Xe light source (full arc) for 2 hours while keeping the reaction at 12 °C using a chiller unit circulating water through a jacket around the reactor. The suspension was then filtered, and the solids were washed with methanol. After this the sample was dried at 60 °C under reduced pressure.

Photocatalysis experiments. Photocatalytic O_2 production was measured in a Pyrex top-irradiation reaction vessel connected to a glass closed gas circulation system. For each experiment photocatalyst (50 mg), water (100 mL) containing $AgNO_3$ (0.01 M), and La_2O_3 (200 mg) were used. The photocatalyst was dispersed using ultrasonication for 10 minutes and the solution was evacuated several times to completely remove air. The reaction was then illuminated with a 300 W Xe light source for the time specified at a fixed distance under reduced pressure. The Xe light source was cooled by water circulating through a metal jacket and the reaction was kept at 12 °C using a chiller unit circulating water through a jacket around the reactor. The head space was analyzed by gas chromatography equipped with a thermal conductivity detector, referencing against standard gases with known concentrations of oxygen.

Transient absorption spectroscopy. The details of the transient absorption spectrometer are reported elsewhere.[8] Briefly, samples were excited with a 400 nm pump pulse at 5 kHz generated using a Pharos-SP-10W (Light Conversion, 1030 nm) operating at 10 kHz coupled to an Orpheus optical parametric amplifier (Light Conversion) in tandem with a Lyra harmonic generator (Light Conversion). The pump power was measured with a power meter (Thor labs) before each measurement and kept at 750 μw. White light generation was achieved by focusing a portion of the Pharos-SP-10W output (10 kHz) onto sapphire within a Harpia-TA spectrometer (Light Conversion). Pump (*ca.* 600 μm diameter) and probe (*ca.* 400 μm) beams were overlapped on the sample which was placed in a quartz cuvette (2 mm). Data were recorded using the Harpia application software and analysed using Carpetview (Light Conversion). TA samples were prepared at the concentrations indicated in the manuscript. Experiments of P10/Co in water with and without degassing using Ar showed no difference (up to 3.4 ns). Attempts to degas P10 and P10/Co in the presence of Ag^+ lead to rapid aggregation and precipitation of the photocatalyst. Therefore, reported experiments in the presence of Ag^+ are carried out in the presence of air without Ar degassing.

Table S1. Optical gap, band positions and oxygen evolution rates (OERs) for the polymer photocatalysts.

Photocatalyst	Optical gap[a] / eV	IP vs SHE[b] / V	EA vs SHE[b] / V	IP* vs SHE[b] / V	EA* vs SHE[b] / V	Contact angle (H_2O) / °	OER[c] / μmol h^{-1}
**P1**	2.78	0.88	-2.33	-2.08	0.64	88	0
**P10**	2.62	1.43	-1.59	-1.44	1.28	59	16.6
**P17**	1.89	0.15	-1.69	-1.66	0.12	77	0
**P24**	2.76	1.05	-2.01	-1.8	0.84	73	1.9
**P26**	3.22	1.22	-2.28	-2.26	1.21	68	0.2
**P28**	2.45	1.17	-1.59	-1.44	1.02	68	4.9
**P29**	2.73	1.35	-1.77	-1.55	1.13	67	0.4
**P30**	2.72	1.17	-1.96	-1.74	0.94	64	0.9
**P31**	2.51	1.22	-1.70	-1.53	1.05	61	1.1
**P35**	2.59	1.02	-1.89	-1.75	0.87	77	1.0

[a] Calculated from the on-set of the absorption spectrum, see discussion in the supporting information; [b] Predicted using (TD-)DFT, values taken from literature, see main text; [c] Reaction conditions: 50 mg polymer photocatalysts loaded with 1 wt. % cobalt was suspended in water/$AgNO_3$/La_2O_3, 300 W Xe light source full arc irradiation.

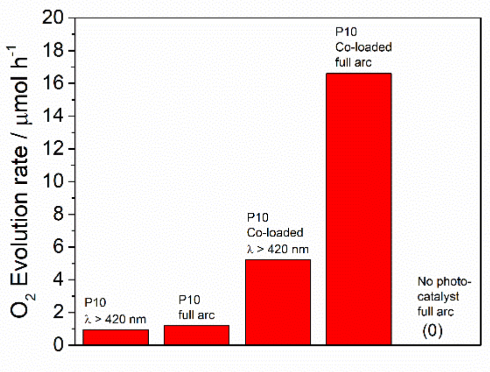

Figure S1. Photocatalytic oxygen evolution of P10 as synthesised and loaded with 1 wt. % cobalt and in absence of a photocatalyst. Conditions: Polymer photocatalyst (50 mg), water (100 mL), $AgNO_3$ (0.01 M), La_2O_3 (200 mg); under either visible light illumination (λ > 420 nm, 300 W Xe light source), or broadband illumination (full arc, 300 W Xe light source).

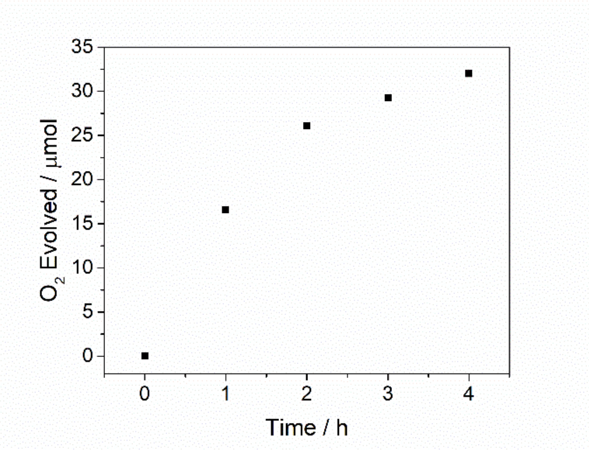

Figure S2. Photocatalytic oxygen evolution of P10 loaded with 1 wt. % cobalt. Conditions: Polymer photocatalyst (50 mg), water (100 mL), $AgNO_3$ (0.01 M), La_2O_3 (200 mg) under broadband illumination (full arc, 300 W Xe light source). Note that the deviation from linearity results from photodeposition of silver metal on the catalyst, which blocks the light.

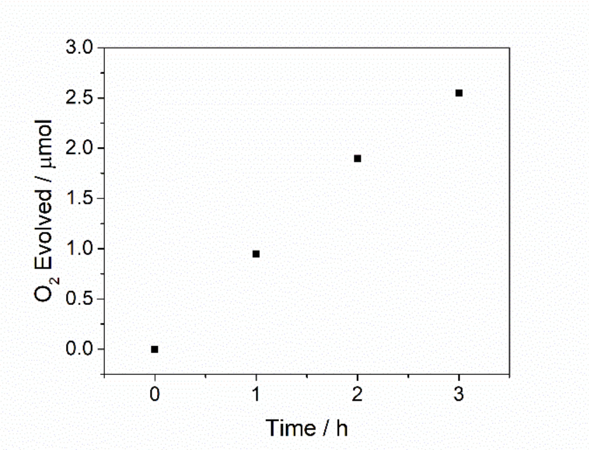

Figure S3. Photocatalytic oxygen evolution of P10 as synthesized. Conditions: Polymer photocatalyst (50 mg), water (100 mL), $AgNO_3$ (0.01 M), La_2O_3 (200 mg) under visible light illumination (λ > 420 nm, 300 W Xe light source).

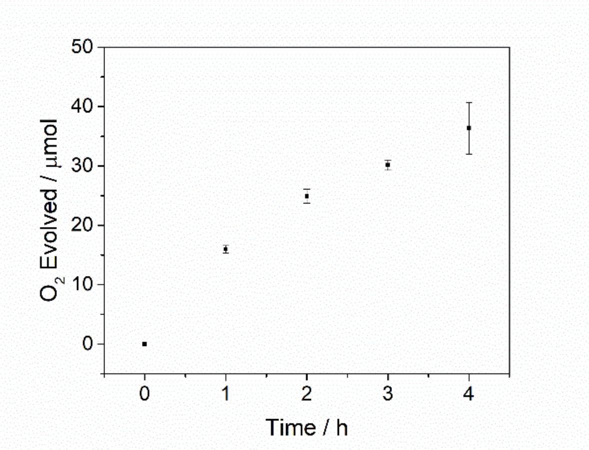

Figure S4. Photocatalytic oxygen evolution of P10 loaded with 1 wt. % cobalt, average of 3 repeat runs. Conditions: Polymer photocatalyst (50 mg), water (100 mL), $AgNO_3$ (0.01 M), La_2O_3 (200 mg) under broadband illumination (full arc, 300 W Xe light source).

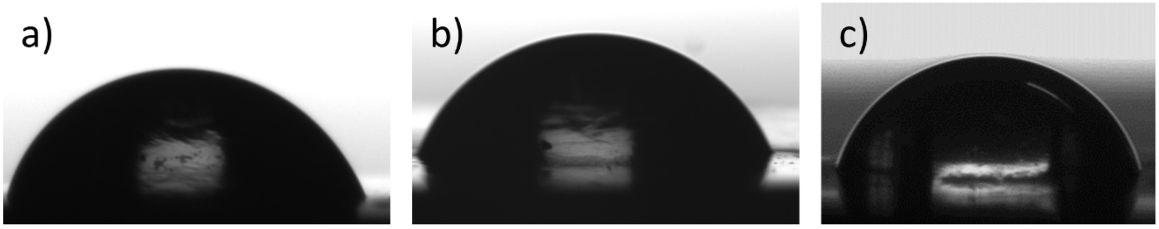

Figure S5. Images of water droplets on the surface of a pressed pallet of a) P17, b) P26, and c) P35.

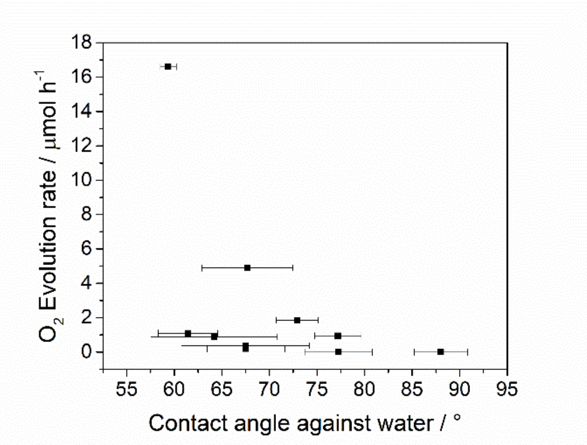

Figure S6. Correlation of the contact angle against water of the ten polymer photocatalysts with the observed oxygen evolution rate under broadband illumination.

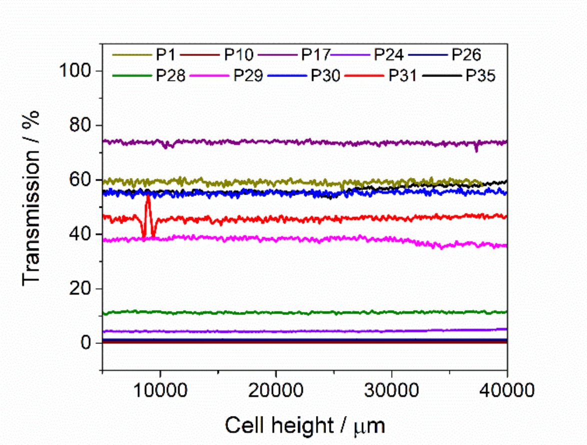

Figure S7. Light obscuration measurements of all polymer photocatalysts dispersed in water measured at 180° relative to the light source (880 nm LED). Low transmittance relates to light being absorbed or scattered.

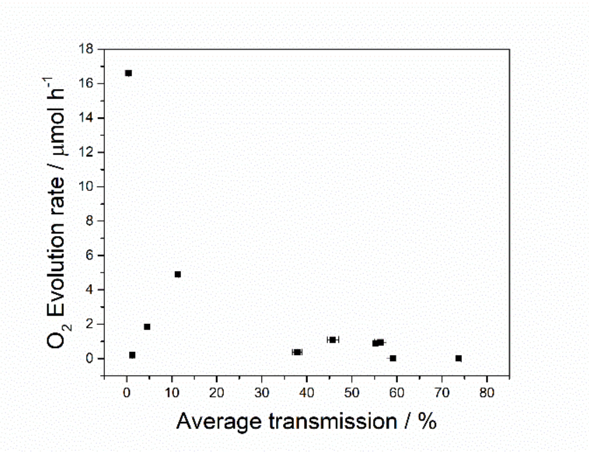

Figure S8. Correlation of the of the average transmission of all polymer photocatalysts in water with the observed oxygen evolution rate under broadband illumination. The transmission is an indirect measure of how well the polymer disperses in the aqueous reaction medium (low transmission = well dispersed).

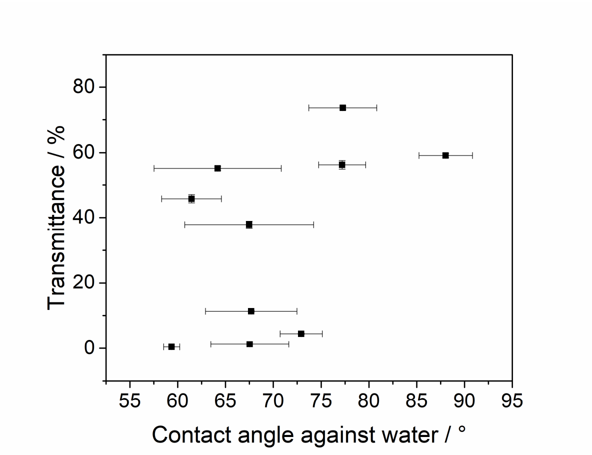

Figure S9. Contact angle of the materials against water of the 10 polymers with their average transmission in water.

Table S2. Average particle sizes and specific surface area as determined from static light scattering experiments.

Photocatalyst	*D*[3,2][a,b]	*D*[4,3][a]	$D_x(50)$ [a]	Specific surface area[a,c] / m^2 kg^{-1}
**P1**	6.21	18.9	16.6	966
**P10**	3.32	7.79	6.50	1806
**P17**	8.52	21.5	18.7	704
**P24**	0.48	17.9	10.7	12,630
**P26**	3.74	6.77	5.10	1603
**P28**	5.56	17.9	15.1	1078
**P29**	2.03	16.4	8.68	2959
**P30**	5.05	16.5	11.3	1189
**P31**	0.64	56.3	29.8	9323
**P35**	4.72	11.4	7.56	1271

[a] Particle sizes as measured by static light scattering on a Mastersizer 3000. [b] Sauter mean diameter (see Equation 1). [c] Relative surface area calculated from the total particle surface area divided by total particle weight assuming a density of 1 g cm^{-3}.

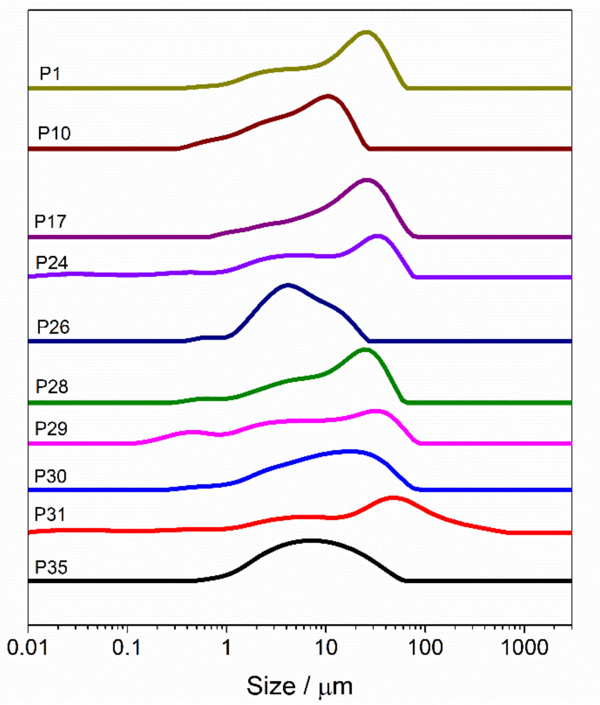

Figure S10. Distribution of particle size of the 10 polymer photocatalysts in water, as determined using static light scattering.

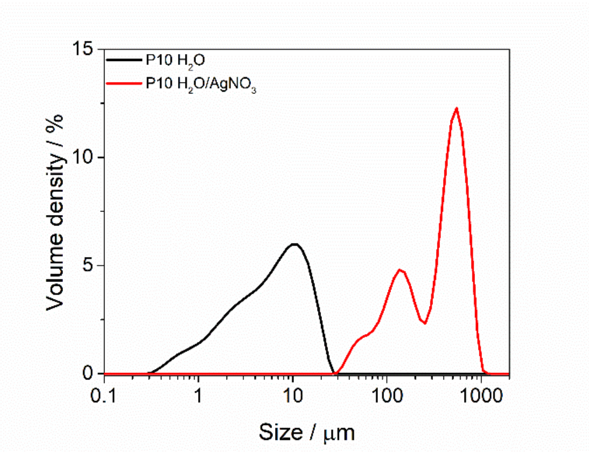

Figure S11. Distribution of particle sizes of P10 in water and in 0.01 M $AgNO_3$ suspension, as determined using static light scattering.

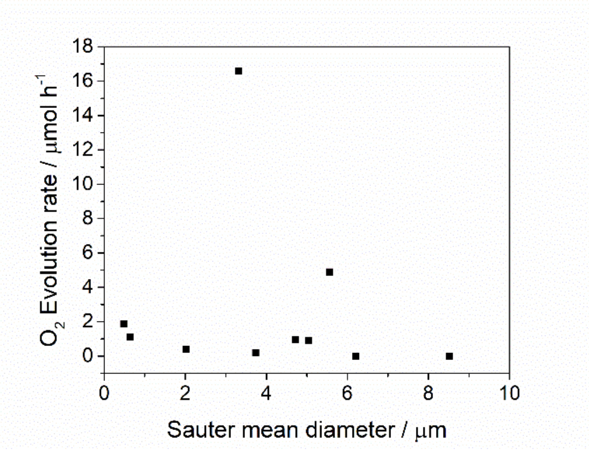

Figure S12. Correlation of the of the Sauter mean diameter of all polymer photocatalysts in water with the observed oxygen evolution rate under broadband illumination.

Table S3. Time correlated single photon counting measurements and results.

Photocatalyst	Environment	λ_{EXC} / nm	λ_{Em} / nm	τ_1[a] / ns	B_1[a]	τ_2[a] / ns	B_2[a]	τ_3[a] / ns	B_3[a]	χ^2	τ_{Avg}[b] / ns
**P1**	Solid-state	375	505	0.25	16.69	0.94	50.13	4.54	33.18	2.08	**2.02**
**Co@P1**	Solid-state	375	505	0.29	18.74	0.94	51.09	4.41	30.18	1.29	**1.86**
**P10**	Solid-state	375	510	0.01	0.01	1.56	67.83	5.36	32.17	1.57	**2.78**
**Co@P10**	Solid-state	375	510	0.41	27.38	0.81	47.69	3.66	24.93	1.24	**1.41**
**P24**	Solid-state	375	500	0.52	33.64	1.57	35.98	4.32	30.38	1.22	**2.05**
**Co@P24**	Solid-state	375	500	0.01	0.85	0.62	43.02	2.94t	56.14	1.11	**1.92**
**P26**	Solid-state	375	490	0.51	43.71	2.06	39.99	8.35	16.30	1.13	**2.41**
**Co@P26**	Solid-state	375	490	0.40	30.53	1.29	36.33	3.86	33.14	1.17	**1.87**
**P28**	Solid-state	375	510	0.39	18.85	1.45	42.20	5.10	38.96	1.14	**2.67**
**Co@P28**	Solid-state	375	510	0.24	22.85	1.48	42.38	5.59	34.77	1.21	**2.62**
**P29**	Solid-state	375	395	0.36	25.55	1.37	41.24	4.39	33.21	1.33	**2.11**
**Co@P29**	Solid-state	375	395	0.03	9.48	0.65	45.40	2.85	45.12	1.12	**1.59**
**P30**	Solid-state	375	490	0.34	22.59	1.34	41.71	5.08	35.70	1.14	**2.45**
**Co@P30**	Solid-state	375	490	0.36	24.18	1.11	36.11	3.81	39.71	1.24	**2.00**
**P31**	Solid-state	375	495	0.45	31.20	1.70	42.05	5.52	26.75	1.23	**2.33**
**Co@P31**	Solid-state	375	495	0.07	3.10	0.61	47.49	2.74	49.41	1.20	**1.65**
**P35**	Solid-state	375	480	0.46	43.02	1.64	37.03	4.85	19.94	1.26	**1.77**
**Co@P35**	Solid-state	375	480	0.29	27.06	1.02	42.89	3.71	30.05	1.18	**1.63**
**Co@P10**	Water[c]	375	510	0.43	10.00	1.02	47.12	3.21	42.88	1.06	**1.90**
**Co@P10**	$AgNO_3$/Water[d]	375	510	0.01	31.23	1.08	27.14	3.42	41.63	1.05	**1.72**

[a] Fluorescence life-times for all polymers in THF suspension obtained from fitting time-correlated single photon counting decays to a sum of three exponentials, which yield τ_1, τ_2, and τ_3 according to $\sum_{i=1}^{n}(A + B_i \exp(-t/\tau_i)$ [b] τ_{AVG} is the weighted average lifetime calculated as $\sum_{i=1}^{n} B_i \tau$ [c] Sample dispersed in water. [d] Sample dispersed in water containing $AgNO_3$ (0.01 M).

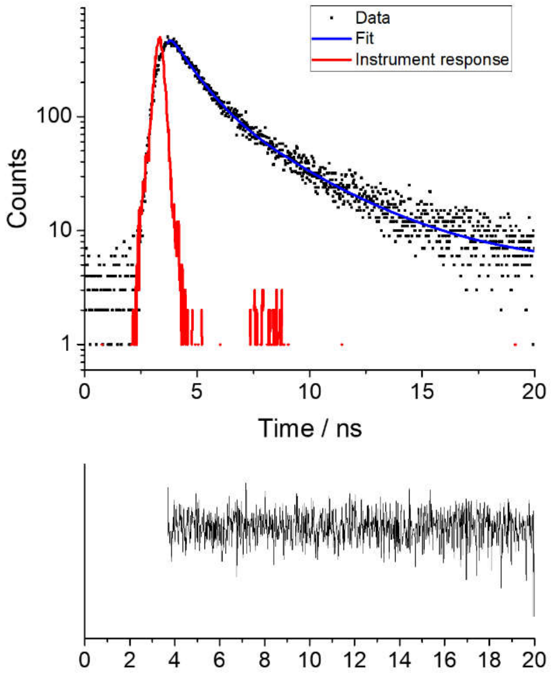

Figure S13. TCSPC data for P10 measured in the solid-state.

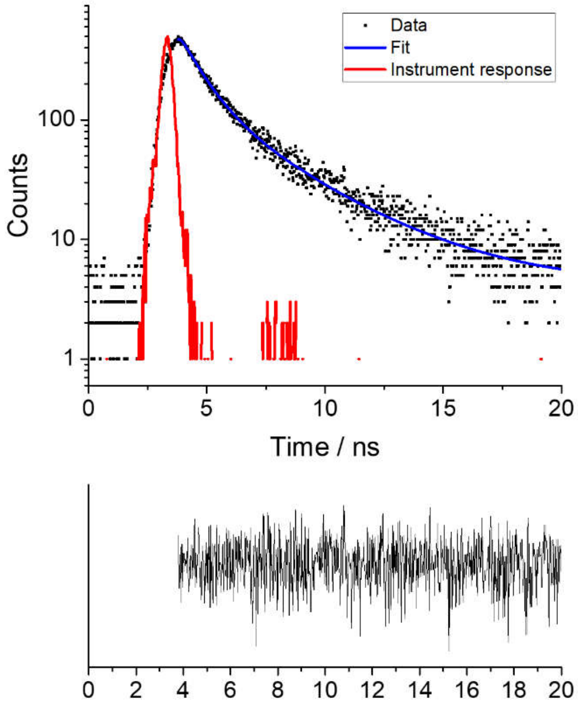

Figure S14. TCSPC data for P10 loaded with cobalt measured in the solid-state.

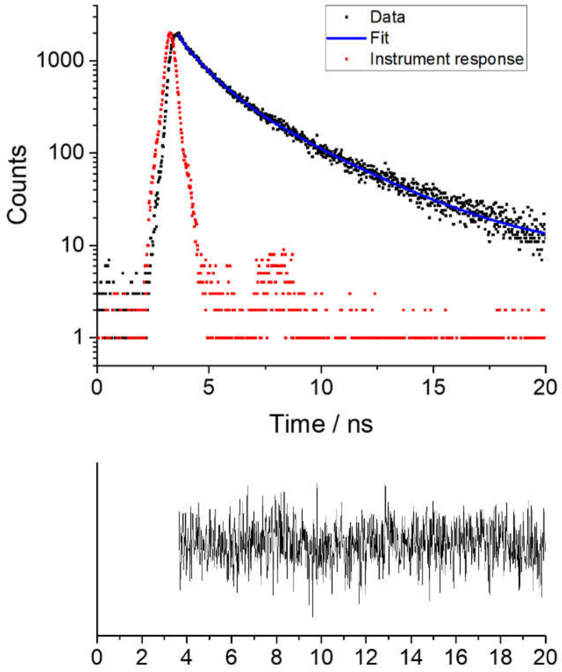

Figure S15. TCSPC data for P10 loaded with cobalt suspended in water.

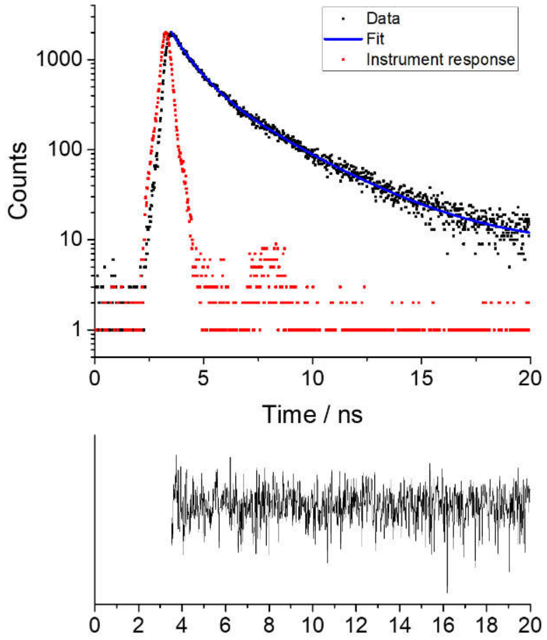

Figure S16. TCSPC data for P10 loaded with cobalt suspended in aqueous $AgNO_3$ solution (0.01 M).

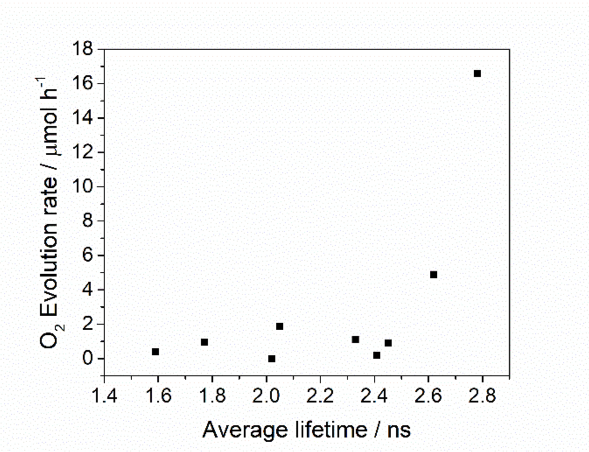

Figure S17. Correlation of the of the average lifetime of all polymer photocatalysts as measured by time-correlated single photon counting with the observed oxygen evolution rate under broadband illumination.

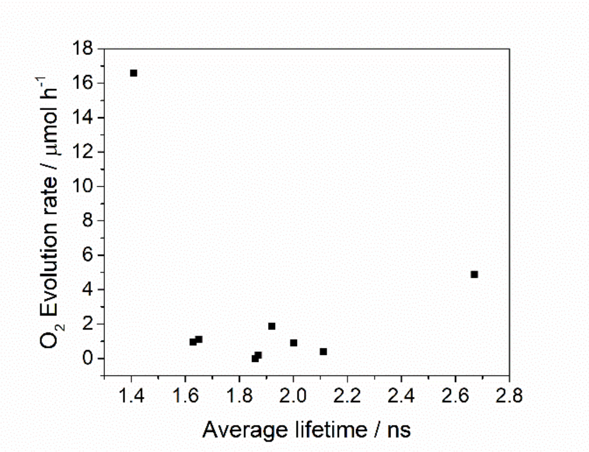

Figure S18. Correlation of the of the average lifetime of all polymer photocatalysts loaded with cobalt as measured by time-correlated single photon counting with the observed oxygen evolution rate under broadband illumination.

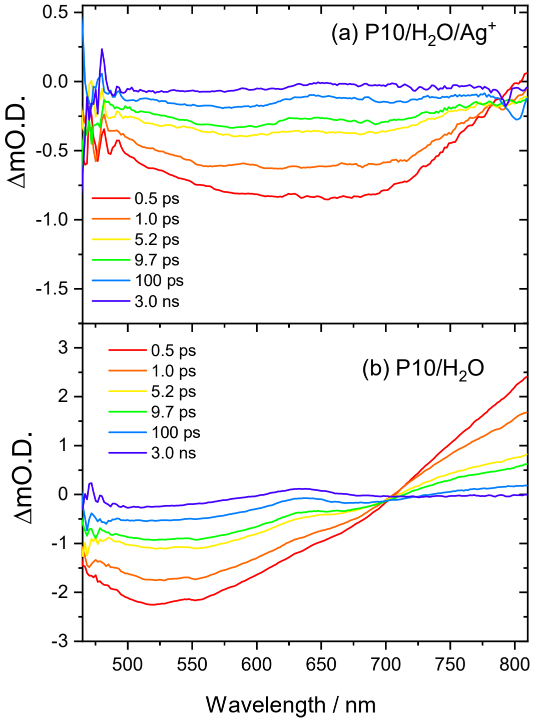

Figure S19. Transient absorption spectra of P10 loaded in water (a) and $AgNO_3$ (0.01 M) (b) following 400 nm (150 nJ pulse, 5 kHz) excitation.

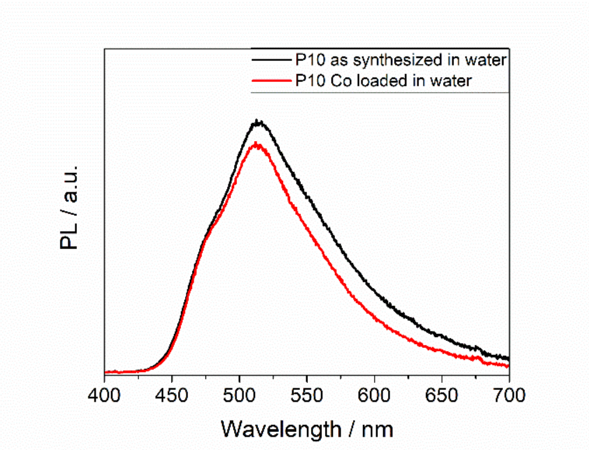

Figure S20. Photoluminescence spectra of P10 measured in water suspension, before and after loading with cobalt.

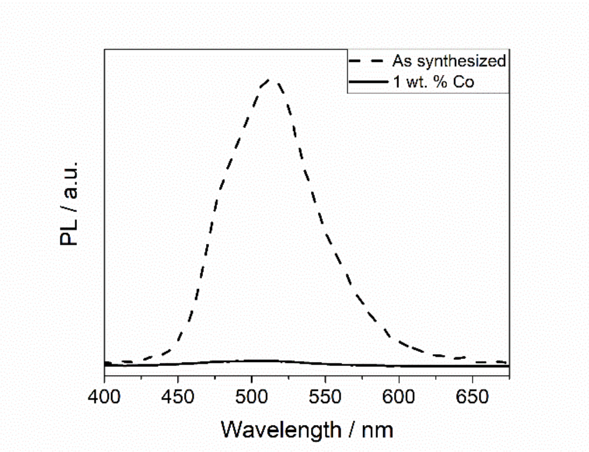

Figure S21. Photoluminescence spectra of P10 measured in the solid state, before and after loading with cobalt.

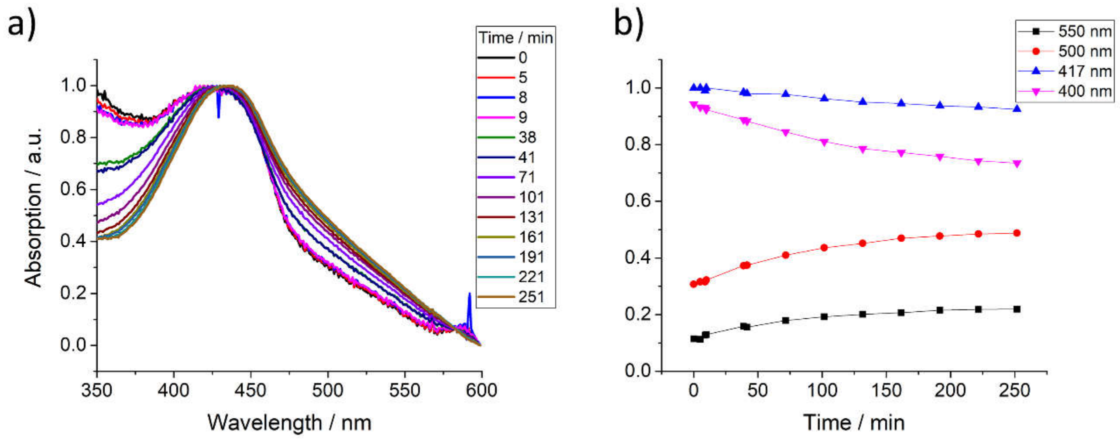

Figure S22. (a) UV-vis measurements over time of P10 in 0.01 M $AgNO_3$ suspension; (b) Change in the UV-vis spectra over time at given wavelength.

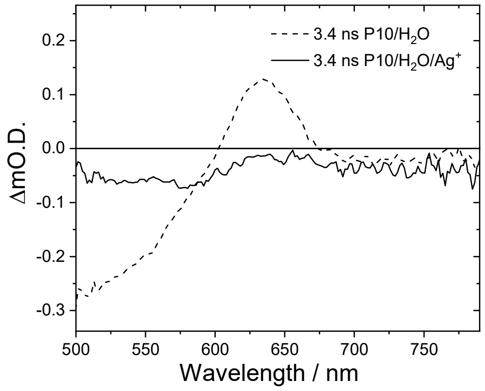

Figure S23. Transient absorption spectra recorded 3.4 ns after excitation (400 nm) of P10 (dotted line) and P10 in the presence of Ag^+ (0.01 M, solid line). The presence of Ag^+ prevents formation of the long-lived 634 nm band assignable to either a partially charge-separated state or the electron polaron.

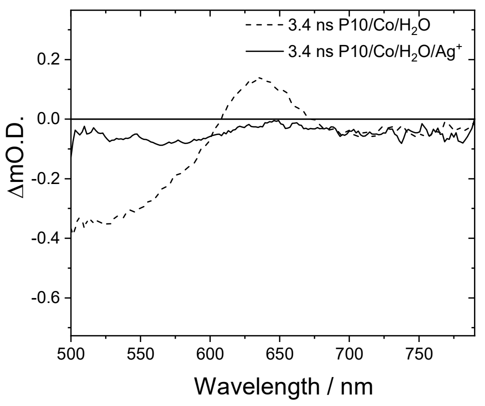

Figure S24. Transient absorption spectra recorded 3.4 ns after excitation (400 nm) of P10/Co (dotted line) and P10/Co in the presence of Ag^+ (0.01 M, solid line). The presence of Ag^+ prevents formation of the long-lived 634 nm band assignable to either a partially charge-separated state or the electron polaron.

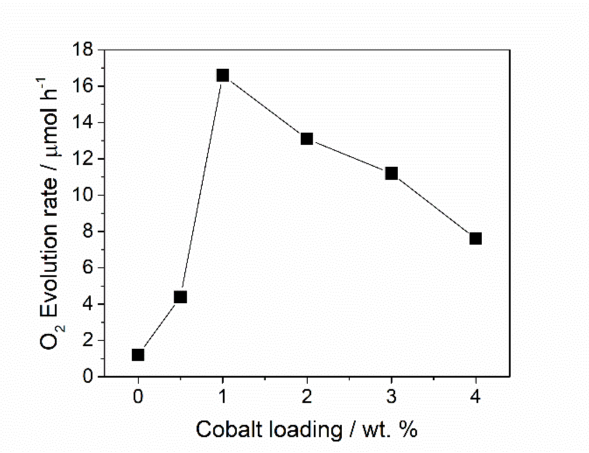

Figure S25. Photocatalytic oxygen evolution of P10 loaded with different amounts of cobalt. Conditions: Polymer photocatalyst (50 mg), water (100 mL), $AgNO_3$ (0.01 M), La_2O_3 (200 mg) under broadband illumination (full arc, 300 W Xe light source).

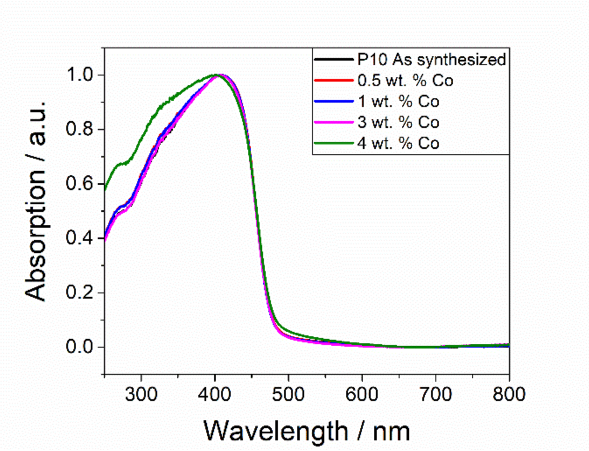

Figure S26. UV-Vis spectra of photocatalyst P10 loaded with different amounts of cobalt.

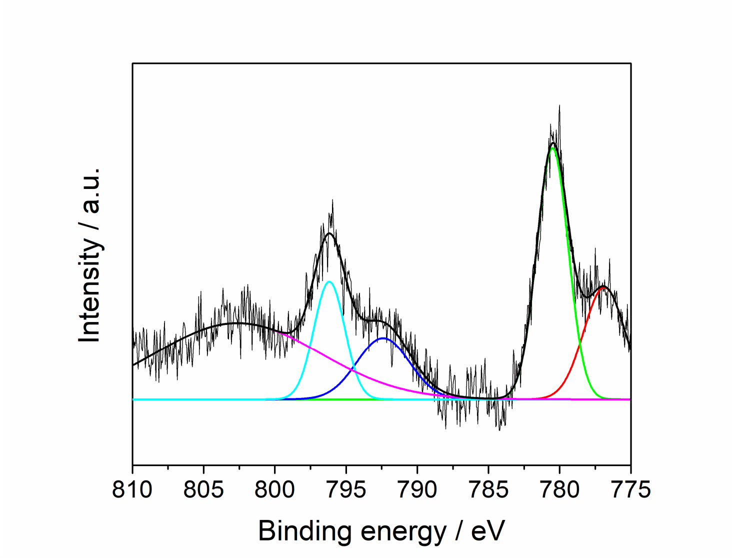

Figure S27. XPS Spectrum in the Co 2p region of P10 after photo-deposition of cobalt.

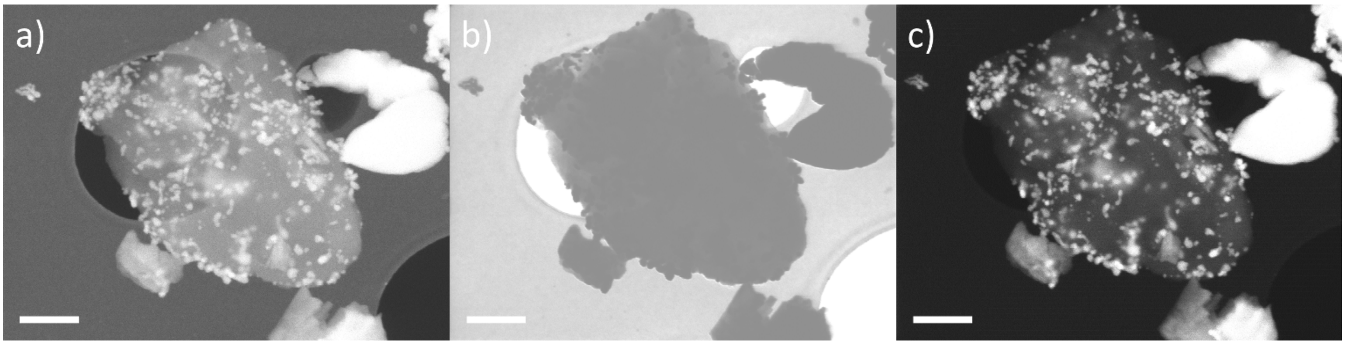

Figure S28. Photocatalyst P10 after 6 hours photocatalysis showing deposition of metallic silver on the surface. (a) SEM image; (b) bright field image; (c) HADF image; The white scale bar is 200 nm.

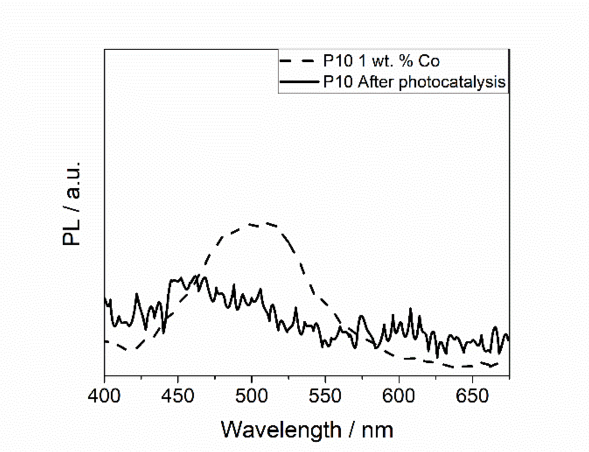

Figure S29. Photoluminescence spectra of P10 measured in the solid-state loaded with cobalt and the material after 6 hours of photocatalytic oxygen production.

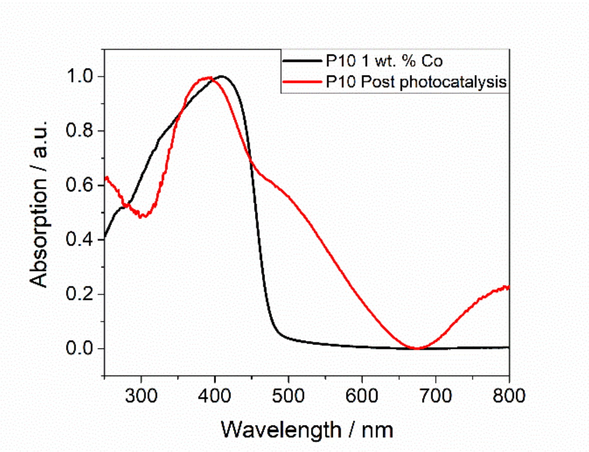

Figure S30. UV-Vis spectra of P10 measured in the solid-state loaded with cobalt and the material after 6 hours of photocatalytic oxygen production.

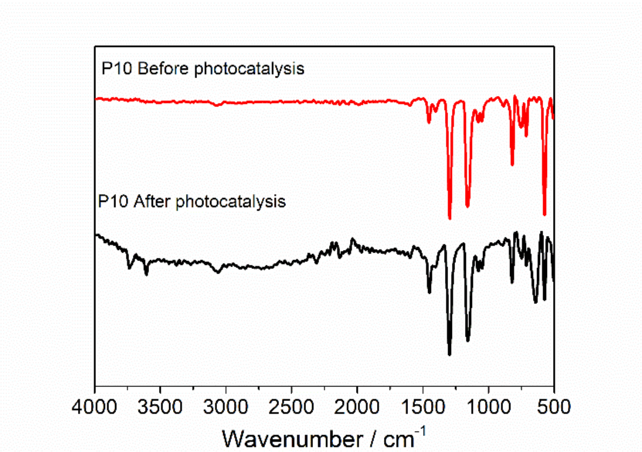

Figure S31. FT-IR spectra of P10 measured as KBr pallets loaded with cobalt and the material after 6 hours of photocatalytic oxygen production.

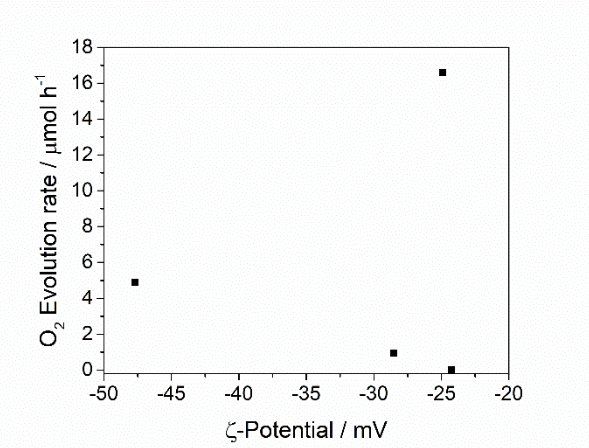

Figure S32. Correlation of the ζ-potentials of P1, P10, P28 and P35 measured in water with the observed oxygen evolution rate under broadband illumination.

References

[1] R. S. Sprick, B. Bonillo, R. Clowes, P. Guiglion, N. J. Brownbill, B. J. Slater, F. Blanc, M. A. Zwijnenburg, D. J. Adams, A. I. Cooper, *Angew. Chem. Int. Ed.* **2016**, *55*, 1792–1796; *Angew.Chem.* **2016**, *128*,1824–1828.

[2] M. Sachs, R. S. Sprick, D. Pearce, S. A. J. Hillman, A. Monti, A. A. Y. Guilbert, N. J. Brownbill, S. Dimitrov, X. Shi, F. Blanc, et al., *Nat. Commun.* **2018**, *9*, 4968.

[3] R. S. Sprick, C. M. Aitchison, E. Berardo, L. Turcani, L. Wilbraham, B. M. Alston, K. E. Jelfs, M. A. Zwijnenburg, A. I. Cooper, *J. Mater. Chem. A* **2018**, *6*, 11994–12003.

[4] R. S. Sprick, L. Wilbraham, Y. Bai, P. Guiglion, A. Monti, R. Clowes, A. I. Cooper, M. A. Zwijnenburg, *Chem. Mater.* **2018**, *30*, 5733–5742.

[5] R. S. Sprick, Y. Bai, A. A. Y. Guilbert, M. Zbiri, C. M. Aitchison, L. Wilbraham, Y. Yan, D. J. Woods, M. A. Zwijnenburg, A. I. Cooper, *Chem. Mater.* **2019**, *31*, 305–313.

[6] M. Kaszube, *Malvern Instruments*, **2017**.

[7] F. Scala, *Fluidized Bed Technologies for Near-Zero Emission Combustion and Gasification*, Woodhead Publishing, **2013**.

[8] Y. Bai, K. Nakagawa, A. J. Cowan, C. M. Aitchison, Y. Yamaguchi, M. A. Zwijnenburg, A. Kudo, R. S. Sprick, A. I. Cooper, *ChemRxiv.* **2020**, DOI:10.26434/chemrxiv.12252317.v1
